# Engineering PE6 prime editors to efficiently insert tags in rice

**DOI:** 10.1111/pbi.14456

**Published:** 2024-09-27

**Authors:** Rongfang Xu, Chong Ma, Jiaqi Sheng, Jiahui Zhu, Dongmei Wang, Xiaoshuang Liu, Qing Wang, Juan Li, Ruiying Qin, Pengcheng Wei

**Affiliations:** ^1^ Anhui Province Key Laboratory of Rice Germplasm Innovation and Molecular Improvement Anhui Academy of Agricultural Sciences Hefei P. R. China; ^2^ College of Agronomy Anhui Agricultural University Hefei P. R. China; ^3^ Research Centre for Biological Breeding Technology, Advance Academy Anhui Agricultural University Hefei P. R. China; ^4^ Wuxi Japonica Rice Industry Technology Center State Key Laboratory of Rice Biology and Breeding & Wuxi Hupper Biological Seed Industry Technology Institute Ltd. Wuxi P. R. China

**Keywords:** prime editing, precise insertion, CRISPR‐Cas, tag, protein labeling

Inserting molecular marker sequences in living plant cells for protein labeling is a great challenge in functional genomic research. We established a simple and easy‐to‐use tag insertion method using an ePE2 system and Genome editing with Reverse transcription templates (RTTs) partially Aligned to each other but Nonhomologous to target sequences within Duo pegRNA (GRAND) strategy (Li *et al*., 2023; Wang *et al*., 2022). Because the insertion efficiency of longer tags, such as 66‐bp 3×FALG, remains insufficient (Li *et al*., 2023), sequential optimizations are urgently needed.

Recently, a series of mammalian PE6 prime editors have been developed for improving efficiency via phage‐assisted evolution and rational design of different reverse transcriptases (RTs) (Doman *et al*., 2023). Among them, PE6c and PE6d exhibited favourable activities at most application scenarios (Doman *et al*., 2023). To construct corresponding plant tools, the RNaseH‐truncated evolved M‐MLV RT of ePE2 was replaced with the *Schizosaccharomyces pombe* Tf1 retrotransposon RT variant to form ePE6c or was introduced the T128N/N200C/V223Y mutations to generate ePE6d (Figure 1a; Supplemental Materials and Methods). To test ePE6s in plants, three epegRNAs were designed for installing small mutations, including a T insertion, a G‐to‐A substitution and a TGTG insertion, in the rice *Pid3*, *Pik‐h* and *TB1* genes, respectively. After *Agrobacterium*‐mediated stable transformation, editing efficiencies were determined in calli using amplicon next‐generation sequencing (NGS). On average, 7.08% and 19.82% of the reads were precisely edited by ePE6c and ePE6d, respectively, showing that both editors are active in rice. In HEK293T cells, the efficiencies of PE6c and PE6d were similar to those of PEmaxΔRNaseH for the installation of point mutations (Doman *et al*., 2023). However, side‐by‐side comparisons showed that the efficiencies of ePE6c were 1.83‐ to 10.12‐fold lower than those of ePE2 (*P* < 0.05, Figure 1b). In contrast, a significant decrease in precise edits by ePE6d was not observed throughout the targets (Figure 1b), while the ratios of the pegRNA scaffold‐derived byproducts of ePE6d were 11.62‐ to 580.98‐fold greater than those of ePE2 at the three sites (*P* < 0.05, Figures S1 and S2). Recent advances indicated that pegRNA scaffold‐derived byproducts could be alleviated by modifying the stem structure of pegRNA (Shuto *et al*., 2024). In this case, we presumed that ePE6d would be as compatible as ePE2 for small edits in plants after further epegRNA optimization.

**Figure 1:**
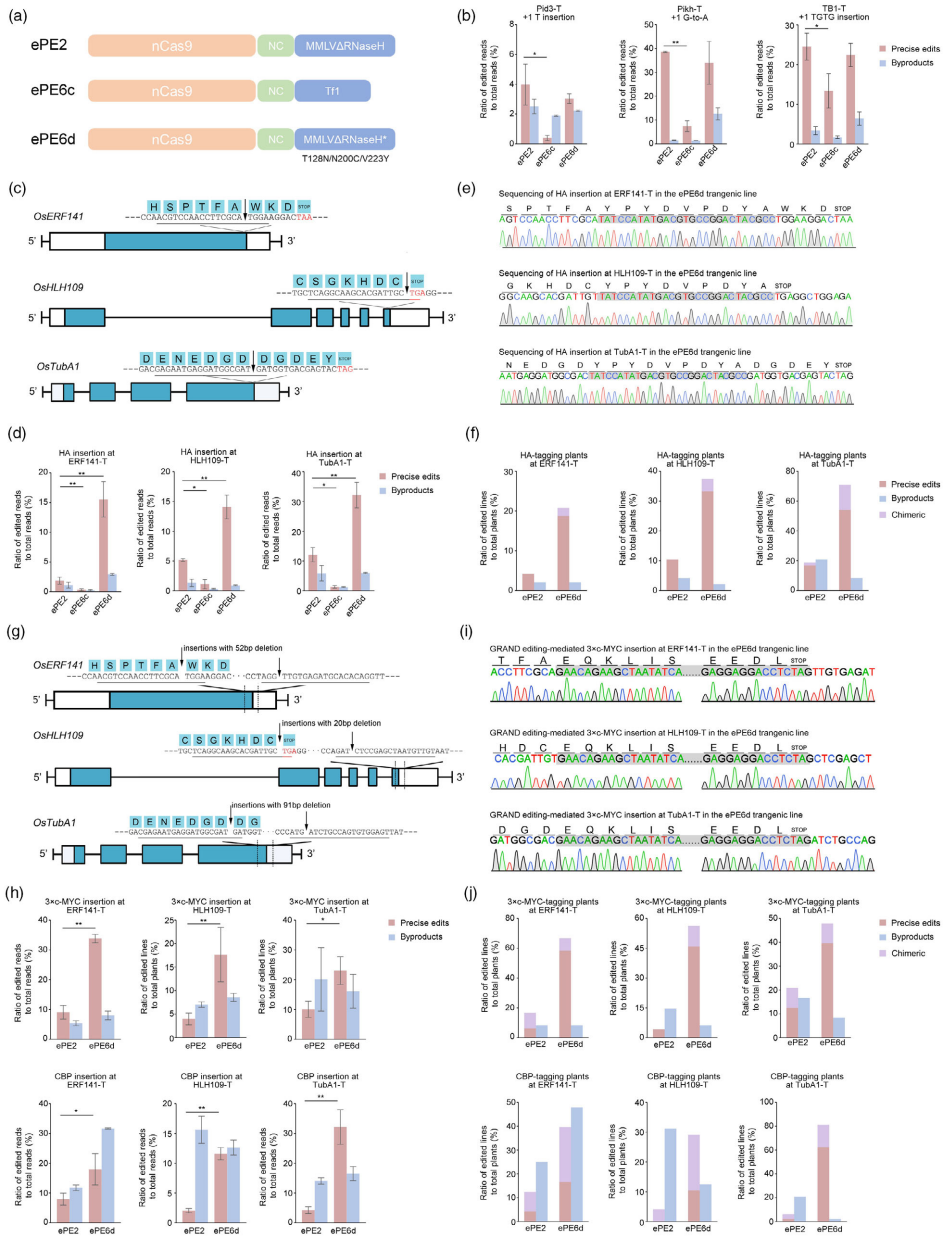
Prime editing of ePE6 variants in rice. (a) Schematic illustration of ePE6 variants. The variants were developed from the ePE2 architecture with engineered SpCas9 nickase (nCas9), RNaseH‐truncated evolved M‐MLV (MMLVΔRNaseH) and an RNA chaperone nucleocapsid (NC) protein. Tf1, the evolved Tf1 retrotransposon RT. MMLVΔRNaseH*, T128N/N200C/V223Y variant of MMLVΔRNaseH. (b) Efficiency of ePE2 and ePE6s for small edits in calli. The sites and types of mutations are indicated. The ratio of precise edit reads (red) or unintended edit reads (blue) to total clean reads was calculated. Independent transformations were performed as biological replicates to determine the mean efficiencies and standard deviations. Differences in the efficiency of precise editing were analysed using two‐tailed *t* tests. *P < 0.05; **P < 0.01. (c) Design of tag knock‐in using a single pegRNA. The protospacers of the pegRNAs are underlined. The insertion sites are indicated by arrows. Red stop codons and bold PAM sites are labelled. (d) Editing efficiency of HA‐tagging with ePE2 and ePE6s in rice calli. (e) Sanger sequencing chromatograms of ePE6d‐mediated HA‐tagged transgenic plants. The insertions are shown in TA clones of the target region. The tag sequences are shadowed. (f) HA‐tagging by ePE2 and ePE6d in T_0_ lines. Knock‐in events were screened using Hi‐TOM analysis from 48 independent lines to calculate the ratio of editing. Plants harbouring precise insertions, byproducts or both types are shown in red, blue and purple, respectively. (g) Design of tag knock‐in with GRAND editing. The lengths of the deleted fragments for GRAND editing are indicated. (h) GRAND editing efficiency of 3×c‐MYC and CBP tagged with ePE2 and ePE6d in rice calli. (i) Sanger sequencing chromatograms of ePE6d‐mediated 3×c‐MYC‐tagged transgenic plants. (j) 3×c‐MYC and CBP tags with ePE2 and ePE6d in the T_0_ lines.

Given that most RT mutations in PE6c and PE6d have evolved for the installation of longer edits (Doman *et al*., 2023), epegRNAs were designed to insert a 27‐bp sequence of the HA tag into the 3′ end of the *OsERF141* (*LOC_Os02g42585*), *OsHLH109* (*LOC_Os01g64780*) and *OsTubA1* (*LOC_Os03g51600*) genes (Figure 1c). Amplicon‐NGS showed that the average HA insertion efficiency of ePE6c was 6.87‐fold lower than that of ePE2 (Figure 1d). Together with the above‐described lower efficiencies of ePE6c for point mutations (Figure 1b), our results suggested that Tf1 RT may be less effective in plants under the current PE architecture. On the other hand, ePE6d generated precise insertions with efficiencies of 15.56%, 14.10% and 32.15% at the ERF141‐T, HLH109‐T and TubA1‐T sites, respectively, with values that were 8.36‐, 2.72‐ and 2.65‐fold greater than those of ePE2 (*P* < 0.05, Figure 1d). The main types of ePE6d byproducts were incomplete insertions followed by short replicates of flanking genome sequences (Figure S3). Along with the enhancement of precise editing activity, the byproduct efficiencies of ePE6d remained at the same level as those of ePE2 at HLH109‐T and TubA1‐T and were slightly increased at ERF141‐T. The insertion of a 30‐bp c‐MYC tag was further examined at the TubA1‐T site. Precise c‐MYC edits were obtained with insignificant different efficiencies by ePE2 and ePE6d (*P* > 0.05, Figure S4), suggesting that ePE6d may be less adaptable for improving the editing of c‐MYC than HA at the same genomic target. It has been reported that the lower structural stability of the pegRNA sequence disrupts PE6d activity (Doman *et al*., 2023). Intriguingly, NUPACK (Fornace *et al*., 2022) prediction demonstrated that the secondary structure of the pegRNA 3′ extension for c‐MYC insertion was more disordered than that for HA (Figure S5), providing a potential clue regarding the behaviour of ePE6d in c‐MYC tag editing. In addition, the tagging vectors were retransformed to assess the activity in the transgenic plants. Precise insertions were induced by ePE6d in 20.83% to 70.83% of the T_0_ lines, which is superior to 4.17% to 18.75% of ePE2 (Figure 1e,f; Table S1). Consistent with the calli results, byproducts occurred with a comparable frequency in 10.94% of ePE2 and 11.46% of ePE6d transgenic plants. Collectively, our data showed that ePE6d outperforms ePE2 for tag insertions in plants.

Next, duo epegRNAs with a 10‐bp RTT overlap were designed to conduct GRAND editing to insert a relatively longer 78‐bp calmodulin‐binding peptide (CBP) tag and 90‐bp 3×c‐MYC tag (Figure 1g). NGS of the callus samples revealed that the CBP insertion of ePE6d in ERF141‐T, HLH109‐T and TubA1‐T increased by 2.26‐, 5.65‐ and 7.56‐fold, respectively, compared with that of ePE2 (*P* < 0.05, Figure 1h). For 3×c‐MYC, ePE6d had a 4.47‐fold improvement in overall efficiency, achieving a maximum editing ratio of 33.84% at ERF141‐T. Moreover, ePE6d offered higher edit:byproduct ratios in five out of the six editing than ePE2 (*P* < 0.05, Figure S6), suggesting a favourable profile for protein tagging. Further validation of the transgenic plants indicated that the average percentage of tagged lines increased from 7.64% of ePE2 to 50% of ePE6d for CBP and from 13.89% of ePE2 to 56.94% of ePE6d for 3×c‐MYC (Figures 1i,j and S7; Table S2). Precise editing occurred maximally in 81.25% of the T_0_ lines for CBP at TubA1‐T and in 66.67% of the T_0_ lines for 3×c‐MYC at ERF141‐T, indicating the outstanding tagging activity of ePE6d. Our previous work showed that ePE2 tagging of 3×FLAG was largely disrupted by incomplete insertions (Li *et al*., 2023). Although partial sequences remained the main unintended edits of ePE6d (Figure S8), no significant increase in byproducts was observed in the examined cases except for the CBP insertion at ERF141‐T. To further demonstrate the compatibility of ePE6d, three additional tags were tested at the TubA1‐T sites. The precise insertion of an 81‐bp 3×HA tag and 135‐bp 3×AVI tag was obtained in 45.83% and 18.75% of the transgenic lines, respectively (Figure S7; Table S2). However, we did not observe the insertion of the intact 171‐bp GB1 tag in the plants, suggesting that the ePE6d knock‐in ability might sharply reduce with size increasing of tags.

Overall, we showed the robust ability of ePE6d to install small edits and to insert approximately 100 bp of epitope tags in rice genome, among which some *in situ* protein labeling was validated using western blotting (Figure S9). We believe that ePE6d is an efficient, versatile and reliable tool for plant gene tagging as well as multiple types of genetic manipulations.

## Author contributions

P.W. and R.Q. supervised the project. P.W. and R.X. designed the experiments and wrote the manuscript with input from all the authors. R.X., C.M., J.Z., D.W. and J.L. optimized the PE vectors and assessed the tagging efficiencies in calli and plants. J.S., X.L. and Q.W. performed *Agrobacterium*‐mediated rice transformation and sampling. R.X., C.M. and J.L. analysed the data.

## Conflict of interest

The authors declare that they have no competing interests.

## Supporting information


**Figures S1–S9** Supplementary Figures.
**Tables S1–S9** Supplementary Tables.

## Data Availability

The data that support the findings of this study are openly available in CRISPR at https://ngdc.cncb.ac.cn/, reference number PRJCA027363.
