# Supplementary material for: Engineering PE6 prime editors to efficiently insert tags in rice
Source: Plant Biotechnol J. 2024 Sep 27;22(12):3383–5. doi: 10.1111/pbi.14456 (PMC11606421; doi:10.1111/pbi.14456)
Supplement: Supplementary file 1 — Figures S1–S9 Supplementary Figures. Tables S1–S9 Supplementary Tables. [file PBI-22-3383-s001.docx]

**Supplemental Materials**

**Supplemental Materials and Methods.**

**Supplemental Figure S1.** Byproduct efficiency for installing small edits with ePE2 and

ePE6s in rice calli.

**Supplemental Figure S2.** Alignments of representative pegRNA scaffold-derived

byproducts in the editing outcomes of ePE6d for installing small edits.

**Supplemental Figure S3.** Alignments of representative editing byproducts of ePE6d

for HA and c-MYC tag insertion.

**Supplemental Figure S4.** Knock-in of the c-MYC tag at the TubA1-T site in rice.

**Supplemental Figure S5.** Structures of pegRNAsequences predicted using NUPACK.

**Supplemental Figure S6.** Edit:byproduct ratios in GRAND editing for tag insertion by

ePE2 and ePE6d.

**Supplemental Figure S7.** Sanger sequencing chromatograms of tags inserted using

ePE6d.

**Supplemental Figure S8.** Alignments of representative byproducts of ePE6d-

mediated GRAND editing for 3×c-MYC insertion.

**Supplemental Figure S9.** Western blotting of CBP-tagged proteins in ePE6d

transgenic plants.

**Supplemental Table S1.** PE-induced tag insertions in T0 transgenic rice.

**Supplemental Table S2.** GRAND editing-induced tag insertions in T0 transgenic rice.

**Supplementary Table S3.** Sequences of the pegRNAs and primers used in this study.

**Supplemental Sequences**

1

**Supplemental Materials and Methods**

Vector construction

For the construction of PE6c, the evolved Tf1 RT sequence was codon-optimized for

rice expression (Supplemental Sequence) and synthesized (GenScript, Nanjing,

China). The nCas9-R221K/N394K-NC sequence was amplified from ePE2 (Li et al.,

2023) and assembled with Tf1 RT using a Gibson Hi-Fi cloning mixture (NEB, Ipswich,

USA). For the construction of ePE6d, the triple T128N/N200C/V223Y mutations were

introduced into the RNaseH-truncated M-MLV RT of ePE2 using a Fast Multisite

Mutagenesis System Kit (TransGen, Beijing, China). In the previously established

pHUC-ePE2 binary vector (Li et al., 2023), the ePE2 fusion fragment was cut by

*Pst*I/*Sac*I double digestion and replaced with ePE6c and ePE6d. Plant PegDesigner

version 1.0 [(http://www.plantgenomeediting.net/)](http://www.plantgenomeediting.net/) and the pegLIT tool

[(http://peglit.liugroup.us)](http://peglit.liugroup.us/) were used to design the PBS sequence with a melting

temperature of 30°C and an 8-nt linker of the RTT for evopreQ1 motif (Lin et al., 2021;

Nelson et al., 2022). The epegRNAs were assembled from separately synthesized

oligos by Golden Gate cloning. The PE binary vectors were predigested with *Bsa*I to

insert epegRNA between the tRNA and hepatitis delta virus (HDV) ribozyme in an

expression cassette driven by a chimeric pol II promoter consisting of a CaMV

enhancer and the CmYLCV promoter. Duo epegRNAs were designed with a 10-bp

overlap at the terminus of RTT for GRAND editing (Wang et al., 2022). The paired

tRNA-epegRNA-HDV modules of each editing were preassembled andsimultaneously

integrated into the expression cassette of the ePE binary vectors. The vector

2

backbones and epegRNAs were validated using Sanger sequencing (Sangon Biotech,

Shanghai, China). The sequences of epegRNAs and primers used are listed in

Supplemental Table S3.

Rice transformation and plant sampling

To perform rice transformation, binary vectors were introduced into the *Agrobacterium*

EHA105 strain using a freeze‒thaw method (Weigel and Glazebrook, 2006). Colony

PCR was applied to identify positive *Agrobacterium* clones, and the results were

confirmed by sequencing the epegRNAregions. Independent clones were selected for

rice transformation as biological replicates. The overnight cultures were suspended to

OD600=0.1 for transfection.

Rice (*Oryza sativa* ssp. *Japonica* cv. Nipponbare) transformation was performed

following a previous protocol with modifications (Hu et al., 2016). Calli were induced

from mature seeds for two to three weeks. Healthy secondary calli were infected with

*Agrobacterium* suspensions for 15 minutes. The transfected calli were allowed to

recover in the dark for 5 days. Approximately 350 to 500 calli were selected under 50

mg/Lhygromycin pressure. Aftertwo to threeweeks ofselection,theefficiency ofprime

editing was assessed in calli. To represent the callus population, only one newly

emerged resistant callus was selected from an independent resistant event, and at

least 200 calli were collected as one sample for efficiency assessments.

For each resistance event, three yellowish, solid, and medium-sized calli were

selected as independent transformation events (originating from one initially infected

callus) for plant regeneration under 25 mg/L hygromycin selection. Only one shoot per

3

transformation event was used for rooting to establish an independent line. Leaves

from three different tillers were used to determine editing in a specific line. All plant

materials were grown at 30°C in a growth chamber under a 16-hour light and 8-hour

dark photoperiod.

Genotyping

Genomic DNA was extracted from the plant samples following a modified

cetyltrimethylammonium bromide (CTAB) protocol (Kuo et al.,2022). Primers designed

on the flanked sequence across target sites were used for genotyping. The NGS

amplicons from the calli samples were produced with Phanta Max Super-Fidelity DNA

Polymerase (Vazyme, Nanjing, China) followed by paired-end sequencing with a PE-

150 pattern using an Illumina NextSeq platform to generate 0.5 GB of dataper sample.

The NGS data are available at the National Genomics Data Center

(https://ngdc.cncb.ac.cn) with accession number PRJCA027363. To assess prime

editing outcomes, the amplicon sequences were aligned with the CRISPResso2

program(Clement et al., 2019). The “Prime editors” model was run for point mutation

editing with a 5-bp pegRNA extension quantification window size, while the “HDR”

model was used for tag insertions with the complete amplicon sequence of the desired

allele as a reference. The editing efficiencies were calculated as previously described

(Anzalone et al., 2019).

The prime editing of transgenic lines was examined using a high-throughput

tracking of mutations (Hi-TOM) assay with a 15% threshold in plants (Liu et al.,2019).

To clearly indicate gene tagging in selected representative lines, Sanger sequencing

4

was performed on TAclones of the target regions.

Western blotting

Protein was extracted from the leaves (0.1 g) of CBP-tagged plants with extraction

buffer containing 100 mM tricin, 10 mM KCl, 1 mM MgCl2, 1 mM

ethylenediaminetetraacetic acid (EDTA), 10% sucrose, 2% Triton-X100, 1 mM DTT,

and 1× protease inhibitor cocktail. The immunoprecipitated samples were separated

using SDS polyacrylamide gel electrophoresis (PAGE) and subjected to immunoblot

analysis with CBP antibodies (Abcam, Cambridge, UK, dilution: 1:10,000).Agoat anti-

mouse or rabbit IgG HRP-conjugated antibody (Sangon Biotech, Shanghai, China,

dilution: 1:10,000) was used as a secondary antibody. After six washes at 7-minute

intervals with TBSplus 0.1% Tween 20, immunoreactiveproteinswerevisualizedusing

SuperSignal West Femto Luminol/Enhancer (Thermo Scientific, Waltham, USA).

Images were captured utilizing a chemiluminescence image analysis system (YPH

Biotech Corporation, Beijing, China).

5

**Supplemental references**

**Anzalone, A.V., Randolph, P.B., Davis, J.R., Sousa, A.A., Koblan, L.W., Levy, J.M., Chen, P.J., Wilson, C., Newby, G.A., Raguram, A., et al.** (2019). Search-and-replace genome editing without double-strand breaks or donor DNA. *Nature* 576:149-157. 10.1038/s41586-019-1711-4.

**Clement, K., Rees, H., Canver, M.C., Gehrke, J.M., Farouni, R., Hsu, J.Y., Cole, M.A., Liu, D.R., Joung, J.K., Bauer, D.E., et al.** (2019). CRISPResso2 provides accurate and rapid genome editing sequence analysis. *Nature Biotechnology* 37:224-226. 10.1038/s41587-019-0032-3.

**Hu, L., Li, H., Qin, R., Xu, R., Li, J., Li, L., Wei, P., and Yang, J.** (2016). Plant phosphomannose isomerase as a selectable marker for rice transformation. *Scientific Reports* 6:25921. 10.1038/srep25921.

**Li, J., Ding, J., Zhu, J., Xu, R., Gu, D., Liu, X., Liang, J., Qiu, C., Wang, H., Li, M., et al.** (2023). Prime editing-mediated precise knockin of protein tag sequences in the rice genome. *Plant Communications* 4: 100572.1016/j.xplc.2023.100572.

**Lin, Q., Jin, S., Zong, Y., Yu, H., Zhu, Z., Liu, G., Kou, L., Wang, Y., Qiu, J.-L., Li, J., et al.** (2021). High-efficiency prime editing with optimized, paired pegRNAs in plants. *Nature Biotechnology* 39:923-927. 10.1038/s41587-021-00868-w.

**Liu, Q., Wang, C., Jiao, X., Zhang, H., Song, L., Li, Y., Gao, C., and Wang, K.** (2019). Hi-TOM: a platform for high-throughput tracking of mutations induced by CRISPR/Cas systems. *Science China Life Sciences* 62:1-7. 1007/s11427-018-9402-9.

**Nelson, J.W., Randolph, P.B., Shen, S.P., Everette, K.A., Chen, P.J., Anzalone, A.V., An, M., Newby, G.A., Chen, J.C., Hsu, A., et al.** (2022). Engineered pegRNAs improve prime editing efficiency. *Nature Biotechnology* 40:402-410. 10.1038/s41587-021-01039-7.

**Kuo, P., Henderson, IR., and Lambing, C.** (2022). CTAB DNA extraction and genotyping-by-sequencing to map meiotic crossovers in plants. *Methods Molecular Biology* 2484: 43-53. 10.1007/978-1-0716-2253-7_4.

**Wang, J., He, Z., Wang, G., Zhang, G., Zhang, R., Duan, J., Gao, P., Lei, X., Qiu, H., Zhang, C., et al.**(2022). Efficient targeted insertionof large DNAfragments without DNAdonors. *Nature Methods* 19:331-340. 10.1038/s41592-022-01399-1.

**Weigel, D., and Glazebrook, J.** (2006). Transformation of agrobacterium using the freeze‒ thaw method. *Cold Spring Harbor Protocols* 2006:pdb. prot4666. 10.1101/pdb.prot4666.

6


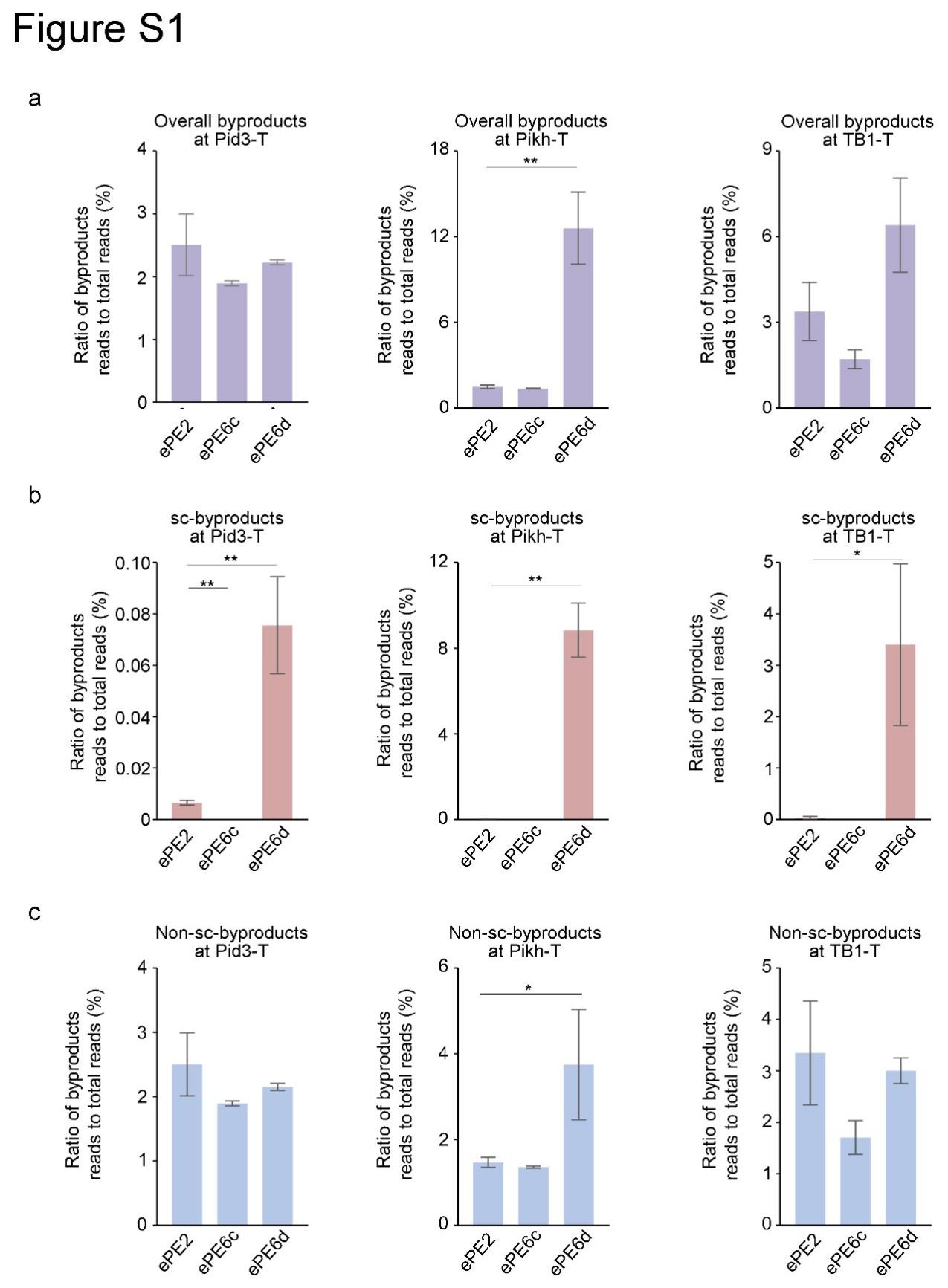


7

**Supplemental Figure S1.** Byproduct efficiency for installing small edits with ePE2 and

ePE6s in rice calli.

The editingbyproducts of ePE2, ePE6candePE6d were comparedatthe three targets.

The ratios were calculated from the reads of overall byproducts (a), pegRNA scaffold-

derived byproducts (sc-byproducts, b) and remaining byproducts (non-sc-byproduct

reads, c) to total clean reads. Independent transformations were performed as

biological replicates to determine the mean efficiencies and standard deviations. The

differences in the efficiencies of the byproducts of ePE2 and ePE6s were analyzed

using two-tailed t tests. *, P<0.05; **, P<0.01.

8


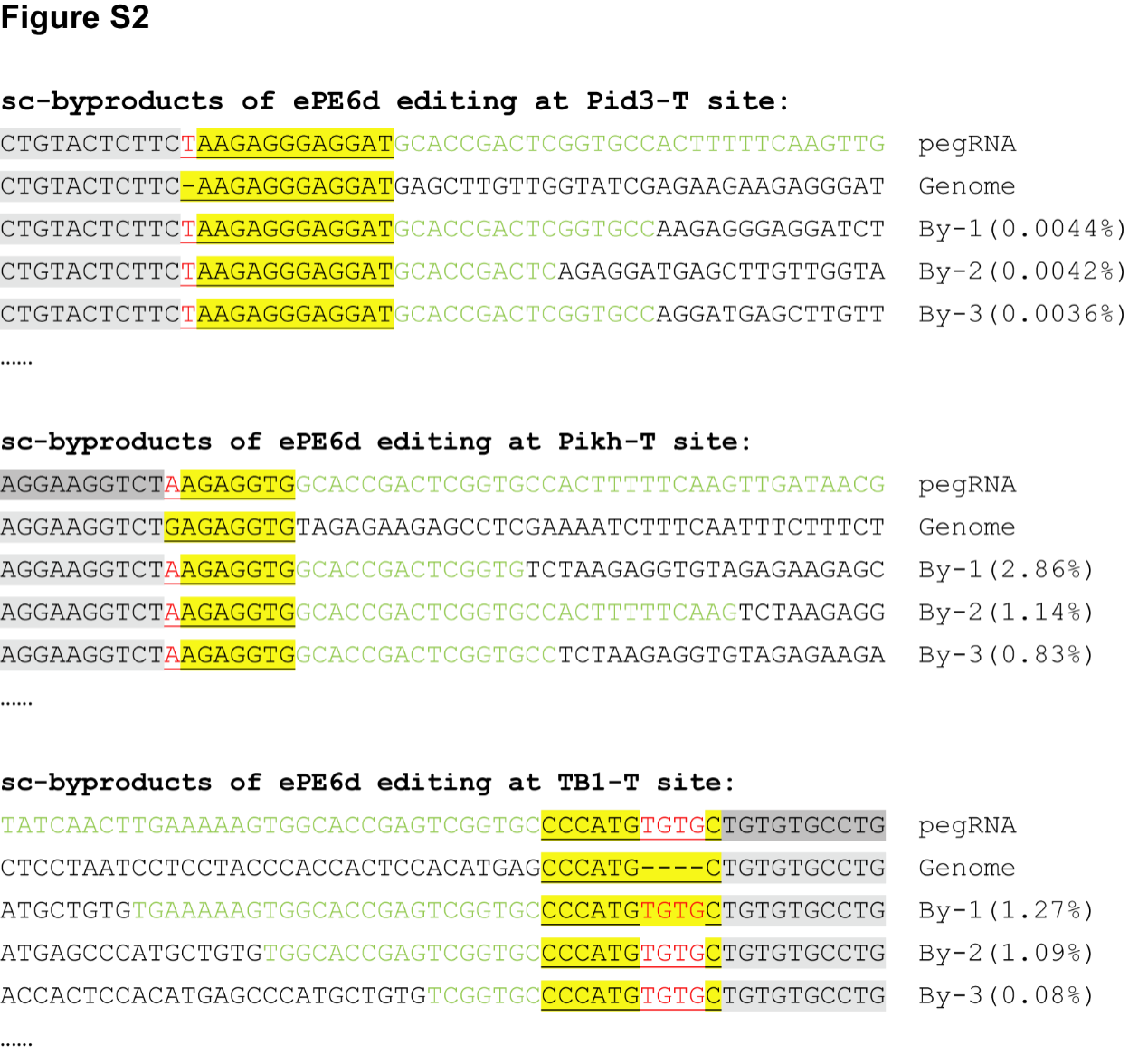


**Supplemental Figure S2.** Alignments of representative pegRNA scaffold-derived

byproducts in the editing outcomes of ePE6d for installing small edits.

The three most frequently occurring pegRNA scaffold-derived byproducts (sc-

byproducts) were obtained from amplicon-NGS and were aligned with pegRNA and

genome sequences. The efficiency was averaged from three biological replicates. The

PBS and RTT sequences are shadowed in gray and yellow, respectively. The desired

mutations are labeled in red. The nucleotides in the byproducts marked with green are

presumed to be derived from the pegRNA scaffold. Notably, random recombination of

the flanking genome may occur along with the insertion of pegRNAscaffolds.

9


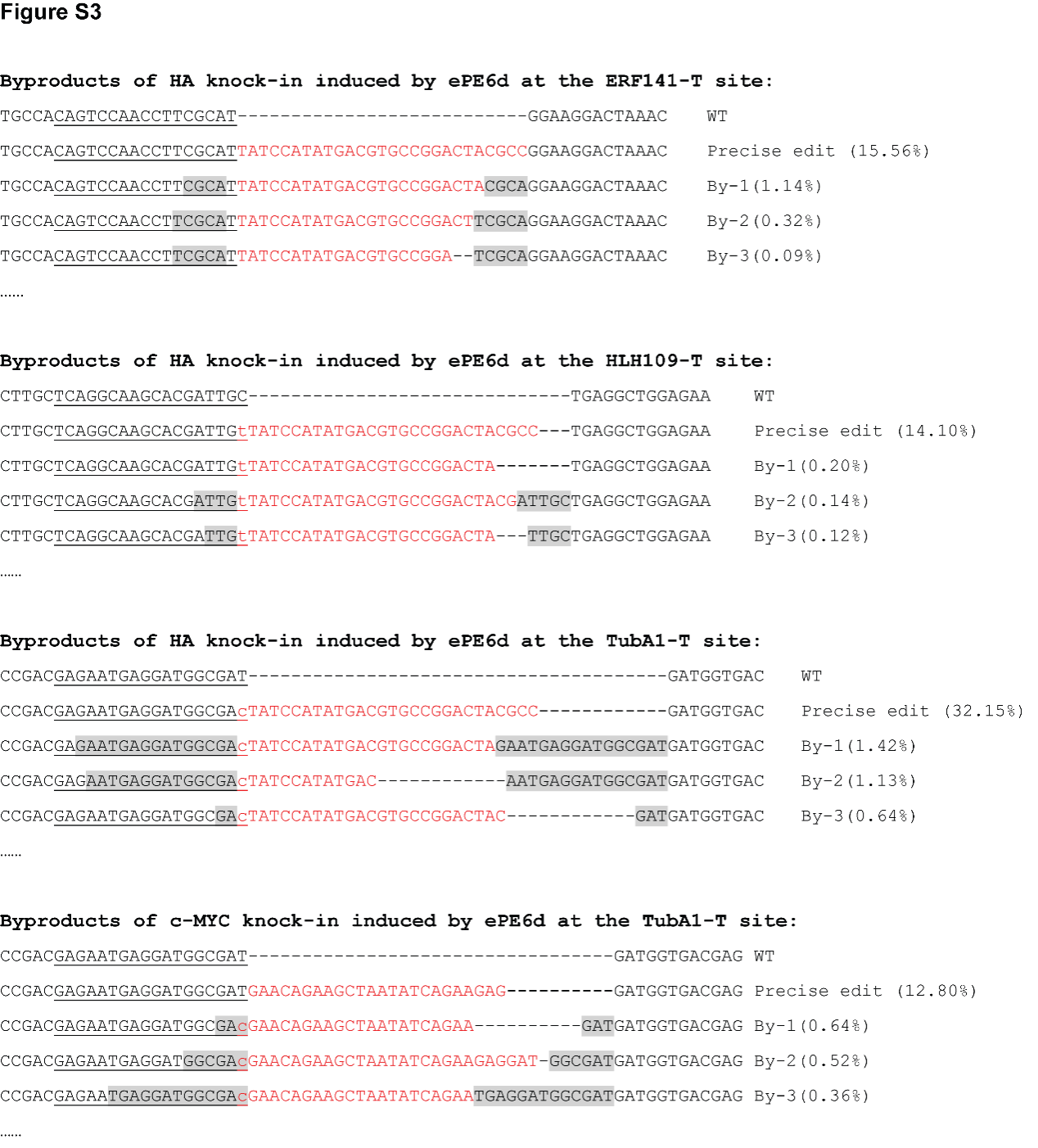


**Supplemental Figure S3.** Alignments of representative editing byproducts of ePE6d

for HA and c-MYC tag insertion.

The three most frequently occurring editing byproducts were obtained from amplicon-

NGS and were aligned with genome sequences and desired edits. The efficiencies

reported in the right brackets are the averages from three biological replicates. The

protospacer sequences of the pegRNAs are underlined. The sequences of tags are

10

labeled in red. The shadow indicates unintended insertion sequences possibly derived

from the flanking genome.

11


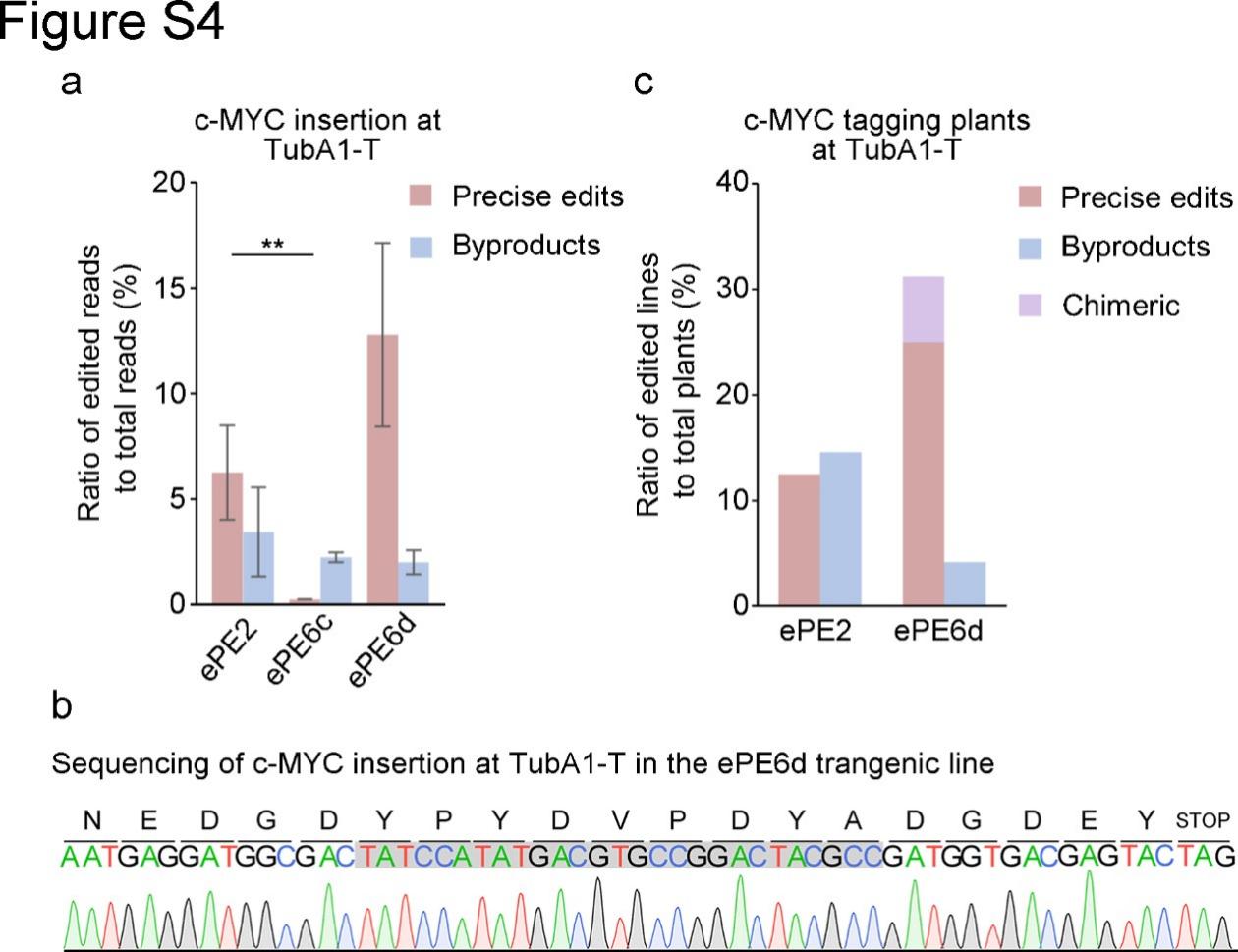


**Supplemental Figure S4.** Knock-in of the c-MYC tag at the TubA1-T site in rice.

a, The efficiencies of ePE2, ePE6c and ePE6d for c-MYC insertion were determined

in rice calli. The ratio of precise edit reads (red) or unintended mutation reads (blue) to

total clean reads was calculated. Independent transformations were performed as

biological replicates to determine the mean efficiencies and standard deviations.

Differences in the efficiency of precise editing were analyzed using two-tailed t tests.

**, P<0.01. b, Sanger sequencing chromatograms of ePE6d-mediated c-MYC tagging

in transgenic plants. The insertions are shown in TA clones of the target region. The

tagsequencesareshadowed. c,c-MYC-tagin T0 lines.Knock-ineventswere screened

using Hi-TOM analysis from 48 independent lines of ePE2 and ePE6d to calculate the

ratio of editing. Plants harboring precise insertions, byproducts or both are shown in

12

red, blue, and purple, respectively.

13


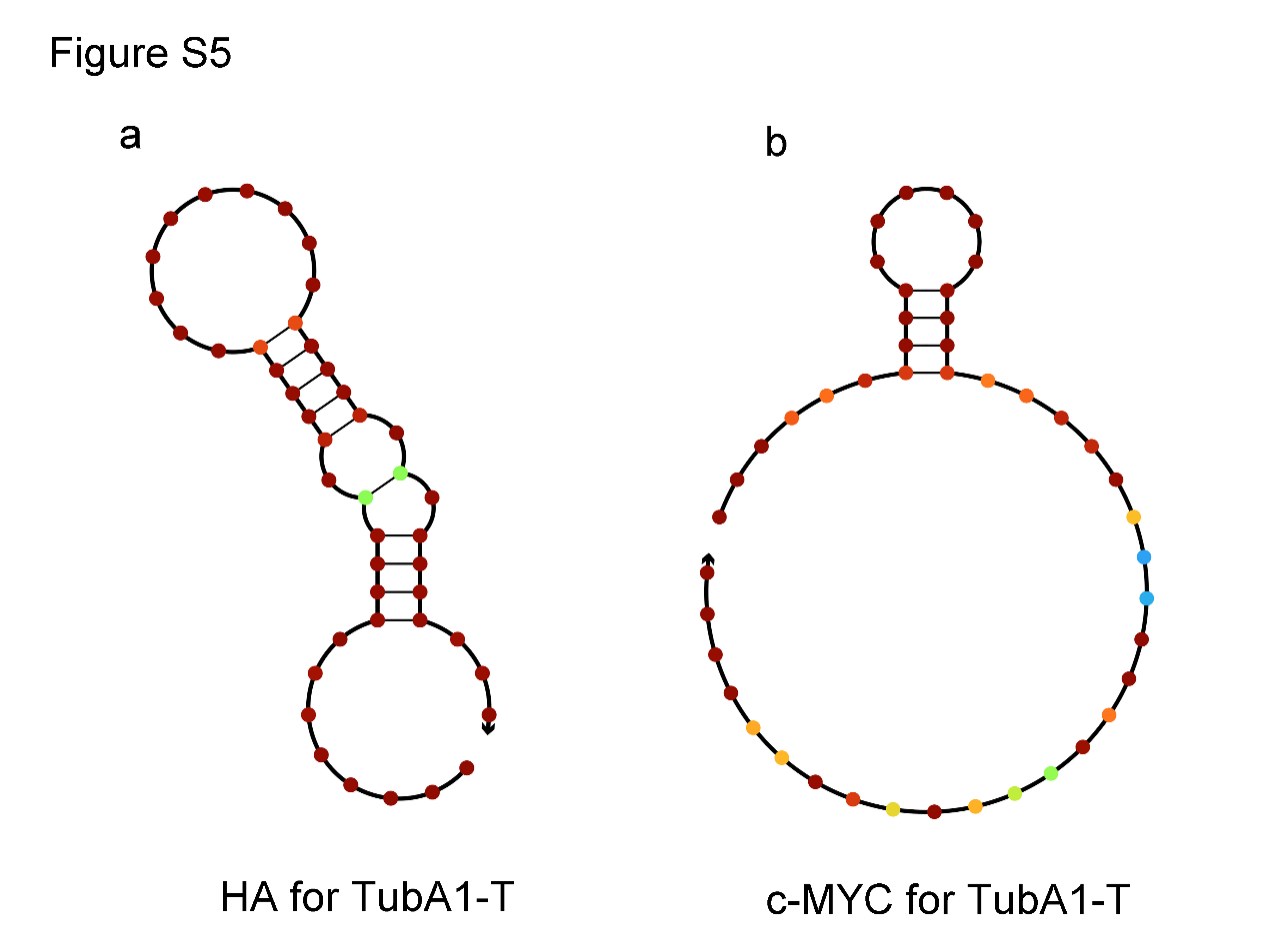


**Supplemental Figure S5.** Structures of pegRNAsequences predicted using NUPACK.

The RTT and PBS extensions of epegRNAs for knock-in of HA (a) and c-MYC (b) at

TubA1-T are shown. The epegRNA structure of the c-MYC tag is largely unpinned.

14


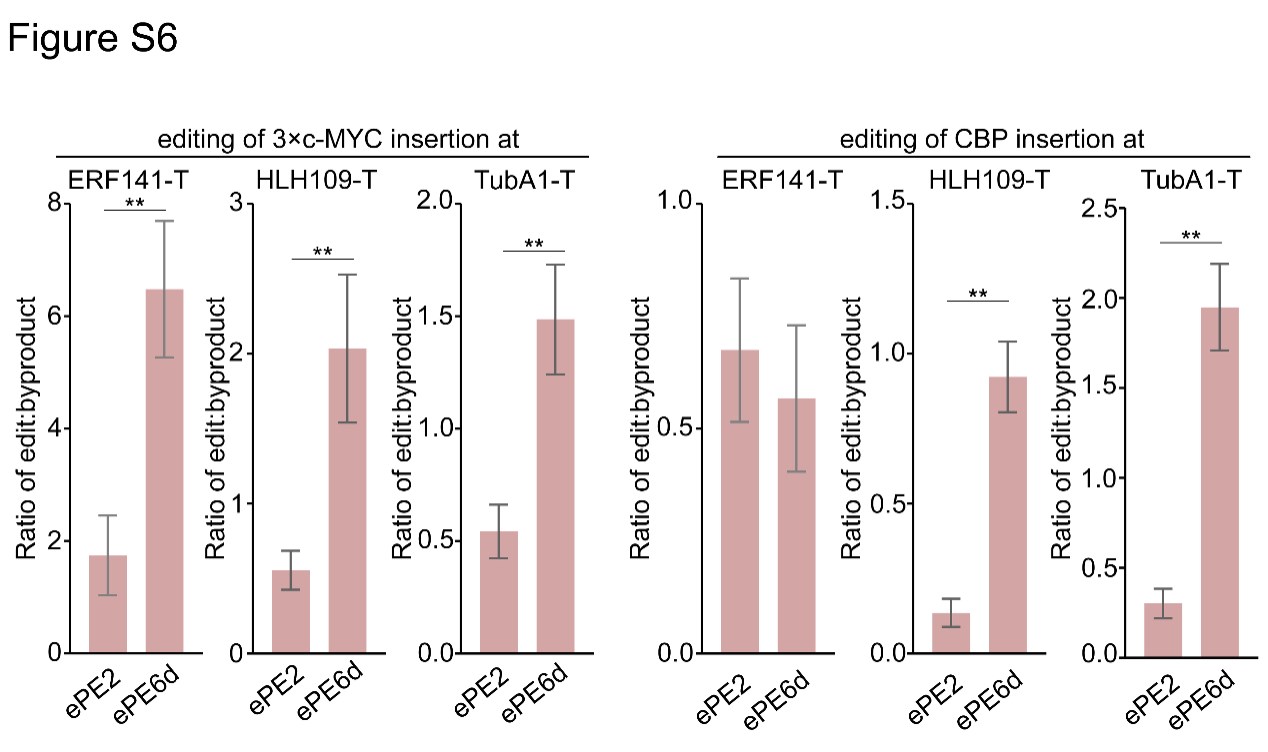


**Supplemental Figure S6.** Edit:byproduct ratios in GRAND editing for tag insertion by

ePE2 and ePE6d.

The ratios were calculated from the efficiencies of edits and byproducts obtained

from the amplicon-NGS of the calli samples. Independent transformations were

performed as biological replicates to determine the mean value. Differences between

ePE2 and ePE6s were analyzed using two-tailed t tests. *, P<0.05; **, P<0.01.

15


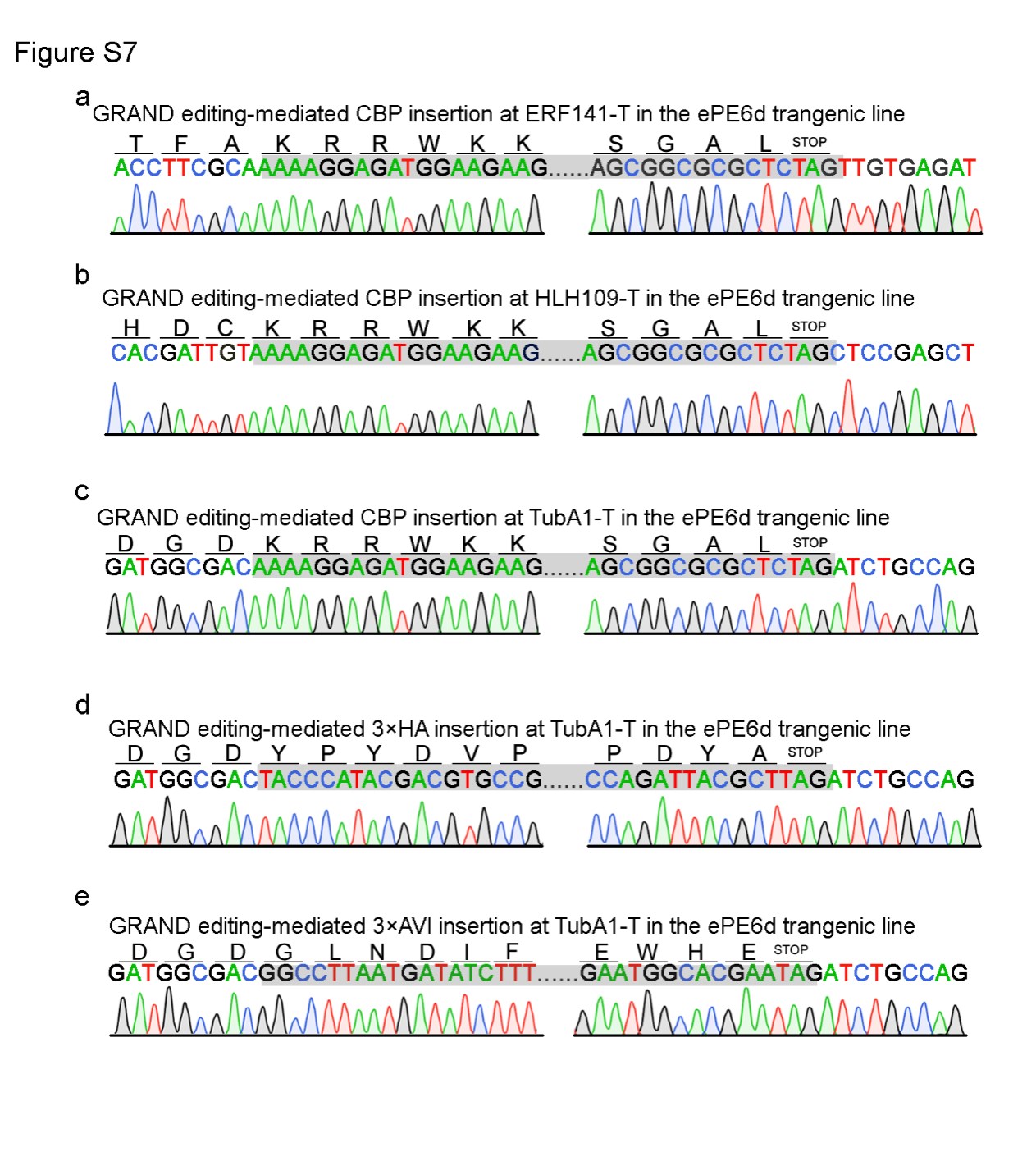


**Supplemental Figure S7.** Sanger sequencing chromatograms of tags inserted using

ePE6d.

The precise insertion of the CBP tag at ERF141-T (a), HLH109-T (b), and TubA1-T

(c); the 3×HA tag at TubA1-T (d); and the 3×AVI tag at TubA1-T (e) in ePE6d

transgenic plants. The TA clones of the target region were subject to Sanger

sequencing. The tag regions are shadowed.

16

**Figure S8**

**Byproducts of 3×c-MYC knock-in induced by ePE6d at the ERF141-T**

TTCGCATGGAAGGACTAAACAGCATCACACGCCACTCTTACACGCAGGAACACCTAGG-----------------------------------------TTGTGAG

TTCGCAGAACAGAAGCTAATATCAGAAGAGGATCTGGAGCAGAAACTGATCAGCGAGGAAGATCTTGAGCAAAAGCTCATCTCCGAGGAGGACCTCTAGTTGTGAG

TTCGCA-----------------------------------------------CGAGGAAGATCTTGAGCAAAAGCTCATCTCCGAGGAGGACCTCTAGTTGTGAG

TTCGCAGAACAGAAGCTAATATCAGAAGAGGATCTGGAG-----------------------------------------------------CCTCTAGTTGTGAG

TTCGCATGGAAGGACTAAACAGCATCACACGCCACTCTTACACGCAGG-------------ATCTTGAGCAAAAGCTCATCTCCGAGGAGGACCTCTAGTTGTGAG

……

**Byproducts of 3×c-MYC knock-in induced by ePE6d at the HLH109-T**

CGATTGCTGAGGCTGGAGAACCAGAT--------------------------------------------------------------------------CTCCGAG

CGATTGtGAACAGAAGCTAATATCAGAAGAGGATCTGGAGCAGAAACTGATCAGCGAGGAAGATCTTGAGCAAAAGCTCATCTCCGAGGAGGACCTCTAGCTCCGAG

CGATTGtGAACAGAAGCTAATATCAGAAGAGGATCT------------------------------------------------------GGACCTCTAGCTCCGAG

CGATTGtGAACAGAAGCTAATATCAGAAGAGGATCTGGAGCAGAAACTGATCAGCGAGGAAGATCTTGAGCAAAAGCTCAT-------------------CTCCGAG

CGATTGtGAACAGAAGCTAATATCAGAAGAGGATCTGGAGCAGAAACTGAT-------------------------------------------------CTCCGAG

……

Genome

Precise edit (33.84%)

By-1(1.51%)

By-2(0.83%)

By-3(0.58%)

Genome

Precise edit (17.66%)

By-1(1.03%)

By-2(0.94%)

By-3(0.59%)

**Byproducts of 3×c-MYC knock-in induced by ePE6d at the TubA1-T**

ATGGCGATGATGGTGACGAGTACTAGAGGAGTCGTCGTCGTCTGGGGGCTTGATGTTCTGTGTGTCAAGGCCTGATTGATAACTGCTGCTATCCCATG----------ATCTGCC

ATGGCGAcGAACAGAAGCTAATATCAGAAGAGGATCTGGAGCAGAAACTGATCA-------GCGAGGAAGATCTTGAGCAAAAGCTCATCTCCGAGGAGGACCTCTAGATCTGCC

ATGGCGATGATGGTGACGAGTACTAGAGGAGTCGTCGTCGTCTGGGGGCTTGATGTTCTGTGCGAGGAAGATCTTGAGCAAAAGCTCATCTCCGAGGAGGACCTCTAGATCTGCC

ATGGCGAcGAACAGAAGCTAATATCAGAAGAGGATCTGGAGCAGAAACTGATCAG-------------------------------------CGAGGAGGACCTCTAGATCTGCC

ATGGCGAcGAACAGAAGCTAATATCAG------------------------------------------------------AAGCTCATCTCCGAGGAGGACCTCTAGATCTGCC

……

Genome

Precise edit (23.08%)

By-1(2.68%)

By-2(2.54%)

By-3(1.69%)

**Supplemental Figure S8.** Alignments of representative byproducts of ePE6d-

mediated GRAND editing for 3×c-MYC insertion.

The three most frequently occurring editing byproducts were obtained by amplicon

NGS and were subsequently aligned with genome sequences and desired edits. For

3×c-MYC editing, the 93-bp insertion contained a 90-bp tag sequence and a 3-bp stop

codon.Theefficiencies intherightbrackets representtheaveragefromthree biological

replicates. The sequences of tags are labeled in red.

18


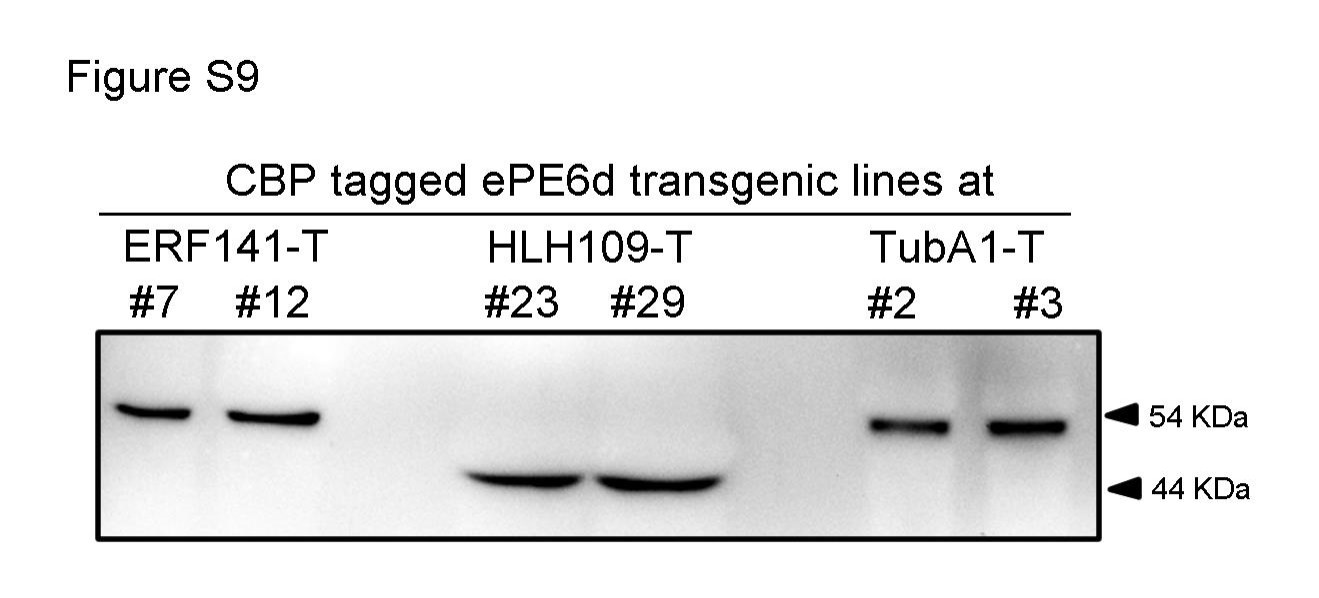


**Supplemental Figure S9.** Western blotting of CBP-tagged proteins in ePE6d

transgenic plants.

PE tagging of *OsERF141*, *OsHLH109*, and *OsTubA1* was examined in representative

T0 transgenic plants with precise edits using an antibody against CBP. The tagged

proteins are indicated with the correct sizes.

19

**Supplemental Table S1.** PE-induced tag insertions in T0 transgenic rice.

Tags Locus Editors Tested plants

Plants carrying precise insertions*

Total (%) Clean edits# (%)

ePE2 ERF141-T ePE6d

ePE2 HA HLH109-T ePE6d

ePE2 TubA1-T ePE6d

ePE2 c-MYC TubA1-T ePE6d

48 02 (04.17%) 48 10 (20.83%) 48 05 (10.42%) 48 18 (37.50%)

48 09 (18.75%) 48 34 (70.83%) 48 06 (12.50%)

48 15 (31.25%)

02 (04.17%) 09 (18.75%) 05 (10.42%) 16 (33.33%)

08 (16.67%) 26 (54.17%)

06 (12.50%)

112 (25.00%)

Individual T0 lines were genotyped by Hi-TOM analysis with a 15% threshold. The frequencies of the mutants are indicated in brackets.

*, the number of plants carrying a precise insertion is indicated.

#, the number of plants carrying precise edits without any byproducts is indicated.

20

**Supplemental Table S2.** GRAND editing-induced tag insertions in T0 transgenic rice.

Tags Locus Editors Tested plants

Plants carrying precise insertions*

Total (%) Clean edits# (%)

3×c-MYC (90 bp)

CBP (78 bp)

3×HA (81 bp) 3×AVI (135 bp)

GB1 (171 bp)

ePE2 ERF141-T ePE6d

ePE2 HLH109-T ePE6d

ePE2 TubA1-T ePE6d

ePE2 ERF141-T ePE6d

ePE2 HLH109-T ePE6d

ePE2 TubA1-T ePE6d

TubA1-T ePE6d TubA1-T ePE6d

TubA1-T ePE6d

48 8 (16.67%) 48 32 (66.67%) 48 02 (04.17%) 48 27 (56.25%)

48 10 (20.83%) 48 23 (47.92%) 48 06 (12.50%) 48 19 (39.58%) 48 02 (04.17%) 48 14 (29.17%) 48 03 (06.25%) 48 39 (81.25%) 48 22 (45.83%) 48 09 (18.75%)

48 00 (00.00%)

03 (06.25%) 28 (58.33%) 02 (04.17%) 22 (45.83%)

06 (12.50%) 19 (39.58%)

02 (04.17%) 08 (16.67%) 00 (00.00%) 05 (10.42%) 01 (02.08%) 30 (62.50%) 07 (14.58%) 04 (08.33%)

00 (00.00%)

Individual T0 lines were genotyped by Hi-TOM analysis with a 15% threshold. The frequencies of the mutants are indicated in brackets.

*, the number of plants carrying a precise insertion is indicated.

#, the number of plants carrying precise edits without any byproducts is indicated.

21

**Supplementary Table S3.** Sequences of the pegRNAs and primers used in this study. 1. epegRNA sequences used in this study

**Target**

Pid3-T

Pikh-T

TB1-T

HA insertion in ERF141-T

HA insertion in HLH109-T

HA insertion in TubA1-T

c-MYC insertion in TubA1-T

3×c-MYC insertion in ERF141-T pegRNA1

3×c-MYC insertion in ERF141-T pegRNA2

3×c-MYC insertion in HLH109-T pegRNA1

3×c-MYC insertion in HLH109-T pegRNA2

3×c-MYC insertion in TubA1-T pegRNA1

3×c-MYC insertion in TubA1-T pegRNA2

CBP insertion in ERF141-T pegRNA1

CBP insertion in ERF141-T pegRNA2

CBP insertion in HLH109-T pegRNA1

CBP insertion in HLH109-T pegRNA2

CBP insertion in TubA1-T pegRNA1

CBP insertion in

**epegRNA Sequence** CTGACTCTGTACTCTTCAAGGTTTCAGAGCTATGCTGGAAACAGCATAGCAAGTTGAAATAAG GCTAGTCCGTTATCAACTTGAAAAAGTGGCACCGAGTCGGTGCATCCTCCCTCTTAGAAGAGT ACAGCCTAAATTT AGCTCGAAGGAAGGTCTGAGGTTTCAGAGCTATGCTGGAAACAGCATAGCAAGTTGAAATAAG GCTAGTCCGTTATCAACTTGAAAAAGTGGCACCGAGTCGGTGCCACCTCTTAGACCTTCCTAA CATAAG CAGACACAGGCACACAGCATGTTTCAGAGCTATGCTGGAAACAGCATAGCAAGTTGAAATAAG GCTAGTCCGTTATCAACTTGAAAAAGTGGCACCGAGTCGGTGCCCCATGTGTGCTGTGTGCCT GCAATTATA CAGTCCAACCTTCGCATGGAGTTTCAGAGCTATGCTGGAAACAGCATAGCAAGTTGAAATAAG GCTAGTCCGTTATCAACTTGAAAAAGTGGCACCGAGTCGGTGCGTCCTTCCAGGCGTAGTCCG GCACGTCATATGGATATGCGAAGGTTATCAAATG TCAGGCAAGCACGATTGCTGGTTTCAGAGCTATGCTGGAAACAGCATAGCAAGTTGAAATAAG GCTAGTCCGTTATCAACTTGAAAAAGTGGCACCGAGTCGGTGCGCCTCAGGCGTAGTCCGGCA CGTCATATGGATAACAATCGTGCCACTTTG GAGAATGAGGATGGCGATGAGTTTCAGAGCTATGCTGGAAACAGCATAGCAAGTTGAAATAAG GCTAGTCCGTTATCAACTTGAAAAAGTGGCACCGAGTCGGTGCTCACCATCGGCGTAGTCCGG CACGTCATATGGATAGTCGCCATCAGAGAAGG GAGAATGAGGATGGCGATGAGTTTCAGAGCTATGCTGGAAACAGCATAGCAAGTTGAAATAAG GCTAGTCCGTTATCAACTTGAAAAAGTGGCACCGAGTCGGTGCACCATCCAGATCCTCTTCTG ATATTAGCTTCTGTTCGTCGCCATCAGAAGAGT CAGTCCAACCTTCGCATGGAGTTTCAGAGCTATGCTGGAAACAGCATAGCAAGTTGAAATAAG GCTAGTCCGTTATCAACTTGAAAAAGTGGCACCGAGTCGGTGCTCGCTGATCAGTTTCTGCTC CAGATCCTCTTCTGATATTAGCTTCTGTTCTGCGAAGGTTGCAGAAGA CTGTGTGCATCTCACAACCTGTTTCAGAGCTATGCTGGAAACAGCATAGCAAGTTGAAATAAG GCTAGTCCGTTATCAACTTGAAAAAGTGGCACCGAGTCGGTGCTGATCAGCGAGGAAGATCTT GAGCAAAAGCTCATCTCCGAGGAGGACCTCTAGTTGTGAGACTTTAAAT TCAGGCAAGCACGATTGCTGGTTTCAGAGCTATGCTGGAAACAGCATAGCAAGTTGAAATAAG GCTAGTCCGTTATCAACTTGAAAAAGTGGCACCGAGTCGGTGCTCGCTGATCAGTTTCTGCTC CAGATCCTCTTCTGATATTAGCTTCTGTTCACAATCGTGCACAATAT ACAACATTAGCTCGGAGATCGTTTCAGAGCTATGCTGGAAACAGCATAGCAAGTTGAAATAAG GCTAGTCCGTTATCAACTTGAAAAAGTGGCACCGAGTCGGTGCTGATCAGCGAGGAAGATCTT GAGCAAAAGCTCATCTCCGAGGAGGACCTCTAGCTCCGAGCACTTATAA GAGAATGAGGATGGCGATGAGTTTCAGAGCTATGCTGGAAACAGCATAGCAAGTTGAAATAAG GCTAGTCCGTTATCAACTTGAAAAAGTGGCACCGAGTCGGTGCTCGCTGATCAGTTTCTGCTC CAGATCCTCTTCTGATATTAGCTTCTGTTCGTCGCCATCGAACCGAA ACTCCACACTGGCAGATCATGTTTCAGAGCTATGCTGGAAACAGCATAGCAAGTTGAAATAAG GCTAGTCCGTTATCAACTTGAAAAAGTGGCACCGAGTCGGTGCTGATCAGCGAGGAAGATCTT GAGCAAAAGCTCATCTCCGAGGAGGACCTCTAGATCTGCCAGCCCTTTAG CAGTCCAACCTTCGCATGGAGTTTCAGAGCTATGCTGGAAACAGCATAGCAAGTTGAAATAAG GCTAGTCCGTTATCAACTTGAAAAAGTGGCACCGAGTCGGTGCCGGCGGACACAGCAATAAAA TTCTTCTTCCATCTCCTTTTTGCGAAGGTTCAAATAAA CTGTGTGCATCTCACAACCTGTTTCAGAGCTATGCTGGAAACAGCATAGCAAGTTGAAATAAG GCTAGTCCGTTATCAACTTGAAAAAGTGGCACCGAGTCGGTGCGTGTCCGCCGCCAACCGCTT CAAGAAAATCTCTTCAAGCGGCGCGCTCTAGTTGTGAGACTAAATTT TCAGGCAAGCACGATTGCTGGTTTCAGAGCTATGCTGGAAACAGCATAGCAAGTTGAAATAAG GCTAGTCCGTTATCAACTTGAAAAAGTGGCACCGAGTCGGTGCCGGCGGACACAGCAATAAAA TTCTTCTTCCATCTCCTTTTACAATCGTGGAGAATAA ACAACATTAGCTCGGAGATCGTTTCAGAGCTATGCTGGAAACAGCATAGCAAGTTGAAATAAG GCTAGTCCGTTATCAACTTGAAAAAGTGGCACCGAGTCGGTGCGTGTCCGCCGCCAACCGCTT CAAGAAAATCTCTTCAAGCGGCGCGCTCTAGCTCCGAGCATCTTTCC GAGAATGAGGATGGCGATGAGTTTCAGAGCTATGCTGGAAACAGCATAGCAAGTTGAAATAAG GCTAGTCCGTTATCAACTTGAAAAAGTGGCACCGAGTCGGTGCCGGCGGACACAGCAATAAAA TTCTTCTTCCATCTCCTTTTGTCGCCATCAAGAGGAG ACTCCACACTGGCAGATCATGTTTCAGAGCTATGCTGGAAACAGCATAGCAAGTTGAAATAAG GCTAGTCCGTTATCAACTTGAAAAAGTGGCACCGAGTCGGTGCGTGTCCGCCGCCAACCGCTT

22

TubA1-T pegRNA2

3×HA insertion in TubA1-T pegRNA1

3×HA insertion in TubA1-T pegRNA2

3×Avi insertion in TubA1-T pegRNA1

3×Avi insertion in TubA1-T pegRNA2

GB1 insertion in TubA1-T pegRNA1

GB1 insertion in TubA1-T pegRNA2

CAAGAAAATCTCTTCAAGCGGCGCGCTCTAGATCTGCCAGATCTGCCAG GAGAATGAGGATGGCGATGAGTTTCAGAGCTATGCTGGAAACAGCATAGCAAGTTGAAATAAG GCTAGTCCGTTATCAACTTGAAAAAGTGGCACCGAGTCGGTGCTGGTACGTCGTAGGGGTAGG CATAATCCGGCACGTCGTATGGGTAGTCGCCATCAATGAACC ACTCCACACTGGCAGATCATGTTTCAGAGCTATGCTGGAAACAGCATAGCAAGTTGAAATAAG GCTAGTCCGTTATCAACTTGAAAAAGTGGCACCGAGTCGGTGCCGACGTACCAGACTACGCGT ATCCGTACGATGTCCCAGATTACGCTTAGATCTGCCAGTAAACTAT GAGAATGAGGATGGCGATGAGTTTCAGAGCTATGCTGGAAACAGCATAGCAAGTTGAAATAAG GCTAGTCCGTTATCAACTTGAAAAAGTGGCACCGAGTCGGTGCAGCTTCGAATATATCGTTGA GTCCCTCGTGCCACTCGATCTTTTGGGCCTCAAAGATATCATTAAGGCCGTCGCCATCGAAGA GAT ACTCCACACTGGCAGATCATGTTTCAGAGCTATGCTGGAAACAGCATAGCAAGTTGAAATAAG GCTAGTCCGTTATCAACTTGAAAAAGTGGCACCGAGTCGGTGCATTCGAAGCTCAGAAAATTG AGTGGCATGAGGGGCTGAACGACATCTTCGAGGCGCAGAAGATTGAATGGCACGAATAGATCT GCCAGTATTTAAA GAGAATGAGGATGGCGATGAGTTTCAGAGCTATGCTGGAAACAGCATAGCAAGTTGAAATAAG GCTAGTCCGTTATCAACTTGAAAAAGTGGCACCGAGTCGGTGCCACCTTTTCAGCGGTGGCGG CGTCTACTGCTTCTGTCGTCGTCTCCCCTTTCAGAGTTTTTCCATTAAGGATGAGCTTGTAGG TGTCCATGTCGCCATCGAAACGAATTGACGCGGTTCTATCTAGTTACGCGTTAAACCAACTAG AAA ACTCCACACTGGCAGATCATGTTTCAGAGCTATGCTGGAAACAGCATAGCAAGTTGAAATAAG GCTAGTCCGTTATCAACTTGAAAAAGTGGCACCGAGTCGGTGCTGAAAAGGTGTTCAAGCAGT ACGCGAACGACAACGGTGTTGATGGCGAGTGGACATATGATGATGCTACCAAGACATTTACTG TCACTGAATAGATCTGCCAGTGATAAGC

23

2. Primers for Hi-TOM analysis of the targets

**Target**

Pid3-T

Pikh-T

TB1-T

ERF141-T

HLH109-T

TubA1-T

**Sequence**

FP: ggagtgagtacggtgtgctaAAATCGATGGCGGGAAGGAGGA

RP: gagttggatgctggatggCATTCCCCACACACTGACCACC FP: ggagtgagtacggtgtgcCCCAAAATTGAGAGCACTGCAT RP: gagttggatgctggatggGGCGATCAAGAGCCTGTAGACC FP: ggagtgagtacggtgtgcTAAGCAACCAAGCACAACCAAA RP: gagttggatgctggatggGCTAGCTAGCTTGGTTGATGCT FP: ggagtgagtacggtgtgcTCCTCCTCGTCCTCCCCCTC RP: gagttggatgctggatggGCTGTCTCAAAAATTTGCAACC FP: ggagtgagtacggtgtgcTTTATGCAGAAGCTAAAACATG RP: gagttggatgctggatggGTTCACCAATCACATCATTACA FP: ggagtgagtacggtgtgcGGAGGTTGGCGCTGAGTCCG

RP: gagttggatgctggatggCACACGCACACGGCAACAGG

24

3. Primers for Sanger sequencing analysis

**Target**

ERF141-T

HLH109-T

TubA1-T

**Sequence**

FP: AACATGGCAGCGACGGCAACAC RP: GCCATCCTTATCCCAGCCTAAA

FP: AGTTGAGGTATGCTAGCTCCAC RP: TTACATACAAGCCACTGCCTTA FP: ATGATCTCCAACTCCACCAGCG

RP: TATTATAAGTGACGAAGCGGTC

25

4. Primers for amplicon deep sequencing

**Target**

Pid3-T

Pikh-T

TB1-T

ERF141-T

HLH109-T

TubA1-T

**Sequence** FP: AAATCGATGGCGGGAAGGAGGA

RP: CATTCCCCACACACTGACCACC

FP: CCCAAAATTGAGAGCACTGCAT

RP: GGCGATCAAGAGCCTGTAGACC

FP: TAAGCAACCAAGCACAACCAAA

RP: GCTAGCTAGCTTGGTTGATGCT

FP: TCCTCCTCGTCCTCCCCCTC

RP: GCTGTCTCAAAAATTTGCAAC

FP: AAAACATGACCAAGCAAACTGC

RP: GCAAAACTTTGGGTTCACCAAT

FP: GGAGGTTGGCGCTGAGTCCG

RP: CACACGCACACGGCAACAGG

26

**Supplemental Sequences** >ePE6c

**Supplemental Sequences**

>ePE6c

**Supplemental Sequences**

>ePE6c

atggccccaaagaagaagcgcaaggtcgacaagaagtactccatcggcctcgacatcggcaccaattctgttggctgggccgtgatcaccgacgagtacaaggtgccgtccaagaagttcaaggtcctcggcaacaccgaccgccactccatcaagaagaatctcatcggcgccctgctgttcgactctggcgagacagccgaggctacaaggctcaagaggaccgctagacgcaggtacaccaggcgcaagaaccgcatctgctacctccaagagatcttctccaacgagatggccaaggtggacgacagcttcttccacaggctcgaggagagcttcctcgtcgaggaggacaagaagcacgagcgccatccgatcttcggcaacatcgtggatgaggtggcctaccacgagaagtacccgaccatctaccacctccgcaagaagctcgtcgactccaccgataaggccgacctcaggctcatctacctcgccctcgcccacatgatcaagttcaggggccacttcctcatcgagggcgacctcaacccggacaactccgatgtggacaagctgttcatccagctcgtgcagacctacaaccagctgttcgaggagaacccgatcaacgcctctggcgttgacgccaaggctattctctctgccaggctctctaagtcccgcaagctcgagaatctgatcgcccaacttccgggcgagaagaagaatggcctcttcggcaacctgatcgccctctctcttggcctcaccccgaacttcaagtccaacttcgacctcgccgaggacgccaagctccagctttccaaggacacctacgacgacgacctcgacaatctcctcgcccagattggcgatcagtacgccgatctgttcctcgccgccaagaatctctccgacgccatcctcctcagcgacatcctcagggtgaacaccgagatcaccaaggccccactctccgcctccatgatcaagaggtacgacgagcaccaccaggacctcacactcctcaaggccctcgtgagacagcagctcccagagaagtacaaggagatcttcttcgaccagtccaagaacggctacgccggctacatcgatggcggcgcttctcaagaggagttctacaagttcatcaagccgatcctcgagaagatggacggcaccgaggagctgctcgtgaagctcaaGagagaggacctcctccgcaagcagcgcaccttcgataatggctccatcccgcaccagatccacctcggcgagcttcatgctatcctccgcaggcaagaggacttctacccgttcctcaaggacaaccgcgagaagattgagaagatcctcaccttccgcatcccgtactacgtgggcccgctcgccaggggcaactccaggttcgcctggatgaccagaaagtccgaggagacaatcaccccctggaacttcgaggaggtggtggataagggcgcctctgcccagtctttcatcgagcgcatgaccaacttcgacaagaacctcccgaacgagaaggtgctcccgaagcactcactcctctacgagtacttcaccgtgtacaacgagctgaccaaggtgaagtacgtgaccgaggggatgaggaagccagctttccttagcggcgagcaaaagaaggccatcgtcgacctgctgttcaagaccaaccgcaaggtgaccgtgaagcagctcaaggaggactacttcaagaaaatcgagtgcttcgactccgtcgagatctccggcgtcgaggataggttcaatgcctccctcgggacctaccacgacctcctcaagattatcaaggacaaggacttcctcgacaacgaggagaacgaggacatcctcgaggacatcgtgctcaccctcaccctcttcgaggaccgcgagatgatcgaggagcgcctcaagacatacgcccacctcttcgacgacaaggtgatgaagcagctgaagcgcaggcgctataccggctggggcaggctctctaggaagctcatcaacggcatccgcgacaagcagtccggcaagacgatcctcgacttcctcaagtccgacggcttcgccaaccgcaacttcatgcagctcatccacgacgactccctcaccttcaaggaggacatccaaaaggcccaggtgtccggccaaggcgattccctccatgaacatatcgccaatctcgccggctccccggctatcaagaagggcattctccagaccgtgaaggtggtggacgagctggtgaaggtgatgggcaggcacaagccagagaacatcgtgatcgagatggcccgcgagaaccagaccacacagaagggccaaaagaactcccgcgagcgcatgaagaggatcgaggagggcattaaggagctgggctcccagatcctcaaggagcacccagtcgagaacacccagctccagaacgagaagctctacctctactacctccagaacggccgcgacatgtacgtggaccaagagctggacatcaaccgcctctccgactacgacgtggacGCtattgtgccgcagtccttcctgaaggacgactccatcgacaacaaggtgctcacccgctccgacaagaacaggggcaagtccgataacgtgccgtccgaagaggtcgtcaagaagatgaagaactactggcgccagctcctcaacgccaagctcatcacccagaggaagttcgacaacctcaccaaggccgagagaggcggcctttccgagcttgataaggccggcttcatcaagcgccagctcgtcgagacacgccagatcacaaagcacgtggcccagatcctcgactcccgcatgaacaccaagtacgacgagaacgacaagctcatccgcgaggtgaaggtcatcaccctcaagtccaagctcgtgtccgacttccgcaaggacttccagttctacaaggtgcgcgagatcaacaactaccaccacgcccacgacgcctacctcaatgccgtggtgggcacagccctcatcaagaagtacccaaagctcgagtccgagttcgtgtacggcgactacaaggtgtacgacgtgcgcaagatgatcgccaagtccgagcaagagatcggcaaggcgaccgccaagtacttcttctactccaacatcatgaatttcttcaagaccgagatcacgctcgccaacggcgagattaggaagaggccgctcatcgagacaaacggcgagacaggcgagatcgtgtgggacaagggcagggatttcgccacagtgcgcaaggtgctctccatgccgcaagtgaacatcgtgaagaagaccgaggttcagaccggcggcttctccaaggagtccatcctcccaaagcgcaactccgacaagctgatcgcccgcaagaaggactgggacccgaagaagtatggcggcttcgattctccgaccgtggcctactctgtgctcgtggttgccaaggtcgagaagggcaagagcaagaagctcaagtccgtcaaggagctgctgggcatcacgatcatggagcgcagcagcttcgagaagaacccaatcgacttcctcgaggccaagggctacaaggaggtgaagaaggacctcatcatcaagctcccgaagtacagcctcttcgagcttgagaacggccgcaagagaatgctcgcctctgctggcgagcttcagaagggcaacgagcttgctctcccgtccaagtacgtgaacttcctctacctcgcctcccactacgagaagctcaagggctccccagaggacaacgagcaaaagcagctgttcgtcgagcagcacaagcactacctcgacgagatcatcgagcagatctccgagttctccaagcgcgtgatcctcgccgatgccaacctcgataaggtgctcagcgcctacaacaagcaccgcgataagccaattcgcgagcaggccgagaacatcatccacctcttcaccctcaccaacctcggcgctccagccgccttcaagtacttcgacaccaccatcgaccgcaagcgctacacctctaccaaggaggttctcgacgccaccctcatccaccagtctatcacaggcctctacgagacacgcatcgacctctcacaactcggcggcgatAGTGGTTCTGAAACTCCTGGAACATCAGAATCGGCGACGCCGGAGAGCGCTACCGTCGTGTCCGGGCAGAAACAAGATAGACAAGGCGGCGAGAGGAGGCGTAGCCAGCTGGACCGCGACCAGTGCGCCTACTGCAAGGAGAAGGGCCACTGGGCAAAAGATTGTCCAAAGAAGCCCCGCGGGCTAAGAGGACCTCGGCCGCAGACCTCCCTCCTTTCAGGCGGCTCCAGCGGCGGCTCTAAGCGGACCGCCGACGGATCAGAGTTCGAGAGCCCGAAGAAGAAGAGGAAGGTGTCCGGCGGCTCATCTGGCGGCTCCATAAGCAGCTCCAAGCACACCCTGTCCCAGATGAACAAGGTGAGCAACATCGTCAAGGAGCCGGAGCTGCCGGACATCTACAAGGAGTTCAAGGACATCACCGCCGACACCAACACCGAGAAGCTCCCGAAGCCGATCAAGGGCCTCGAGTTCGAGGTGGAGCTGACCCAGGAGAACTACCGCCTCCCGATCCGCAACTACCCGCTGacaCCGgtgAAGATGCAGGCCATGAACGACGAGATCAACCAGGGCCTCAAGggcGGCATCATCCGGGAGTCCAAGGCCATCAACGCGTGCCCGGTCATcTTCGTGCCGcgcAAGGAGGGCACCCTCAGGATGGTGGTGGACTACAgGCCGCTGAACAAGTATGTGAAGCCGAACgTgTACCCGCTCCCGCTGATCGAGCAGCTCCTCGCCAAGATCCAGGGCTCTACCATCTTCACCAAGCTGGATCTCAAGTCCGCGTACCACCagATCCGCGTGAGGAAGGGCGACGAGCACAAGCTGGCCTTCAGGTGCCCACGCGGCGTCTTTGAGTACCTCGTGATGCCGTACGGCATCaagACCGCCCCGGCCCACTTCCAGTACTTCATCAACACCATCCTCGGCGAGGCCAAGGAGTCCCATGTGGTGTGCTACATGGATGACATCCTCATCCACAGCAAGTCCGAGTCTGAGCACGTGAAGCACGTGAAGGACGTGCTCCAGAAGCTCAAGAACGCCAACCTCATCATCAACCAGGCCAAGTGCGAGTTCCACCAGTCCCAGGTGAAGTTCcTCGGCTACCACATCTCCGAGAAGGGCcTCACCCCATGCCAGGAGAACATCGACAAGGTGCTCCAGTGGAAGCAGCCGAAGAACCagAAGGAGCTGAGGCAGTTCCTCGGCcagGTGAACTACCTCCGCAAGTTCATCCCCAAGACCTCCCAGCTCACCCACCCGCTCAACAAGCTCCTCAAGAAGGACGTGCGCTGGAAGTGGACCCCGACCCAGACCCAGGCCATCGAGAACATCAAGCAGTGCCTCGTGAGCCCGCCGGTGCTGAGGCACTTCGATTTCAGCAAGAAGATCCTGCTCGAGACTGACGtgAGCGACGTGGCGGTGGGCGCCGTGCTCTCCCAGAAGCACGACGACGATAAGTACTACCCGGTGGGCTACTACAGCGCCAAGATGAGCAAGGCCCAGCTCAACTACTCCGTGTCCGACAAGGAGATGCTCGCCATCATCAAGTCCCTCgAGCACTGGCGCCACTACCTCGAGTCCACCATCGAGCCGTTCAAGATCCTCACCGACCACAGGAACCTCATCGGCCGCATCACCAACGAGTCCGAGCCGGAGAACAAGCGCCTCGCCCGCTGGCAGCTGTTCCTCCAGGACTTCAACTTCGAGATCAACTACAGGCCGGGCTCCGCCAACCACATCGCCGACGCCCTCAGCCGCATCGTGGATGAGACTGAGCCGATCCCGAAGGACAaCGAGGACAACAGCATCAACTTCGTGAACCAGATCAGCATCAGCCCGAAGAAGAAGAGGAAAGTGGGATCAGGACCAGCCGCCAAGAGGGTGAAGCTCGATTAG

Blue: SpCas9 nickase;

Red: NC peptide;

Green: Tf1 RT variant;

Yellow: NLS.

>ePE6d

atggccccaaagaagaagcgcaaggtcgacaagaagtactccatcggcctcgacatcggcaccaattctgttggctgggccgtgatcaccgacgagtacaaggtgccgtccaagaagttcaaggtcctcggcaacaccgaccgccactccatcaagaagaatctcatcggcgccctgctgttcgactctggcgagacagccgaggctacaaggctcaagaggaccgctagacgcaggtacaccaggcgcaagaaccgcatctgctacctccaagagatcttctccaacgagatggccaaggtggacgacagcttcttccacaggctcgaggagagcttcctcgtcgaggaggacaagaagcacgagcgccatccgatcttcggcaacatcgtggatgaggtggcctaccacgagaagtacccgaccatctaccacctccgcaagaagctcgtcgactccaccgataaggccgacctcaggctcatctacctcgccctcgcccacatgatcaagttcaggggccacttcctcatcgagggcgacctcaacccggacaactccgatgtggacaagctgttcatccagctcgtgcagacctacaaccagctgttcgaggagaacccgatcaacgcctctggcgttgacgccaaggctattctctctgccaggctctctaagtcccgcaagctcgagaatctgatcgcccaacttccgggcgagaagaagaatggcctcttcggcaacctgatcgccctctctcttggcctcaccccgaacttcaagtccaacttcgacctcgccgaggacgccaagctccagctttccaaggacacctacgacgacgacctcgacaatctcctcgcccagattggcgatcagtacgccgatctgttcctcgccgccaagaatctctccgacgccatcctcctcagcgacatcctcagggtgaacaccgagatcaccaaggccccactctccgcctccatgatcaagaggtacgacgagcaccaccaggacctcacactcctcaaggccctcgtgagacagcagctcccagagaagtacaaggagatcttcttcgaccagtccaagaacggctacgccggctacatcgatggcggcgcttctcaagaggagttctacaagttcatcaagccgatcctcgagaagatggacggcaccgaggagctgctcgtgaagctcaaGagagaggacctcctccgcaagcagcgcaccttcgataatggctccatcccgcaccagatccacctcggcgagcttcatgctatcctccgcaggcaagaggacttctacccgttcctcaaggacaaccgcgagaagattgagaagatcctcaccttccgcatcccgtactacgtgggcccgctcgccaggggcaactccaggttcgcctggatgaccagaaagtccgaggagacaatcaccccctggaacttcgaggaggtggtggataagggcgcctctgcccagtctttcatcgagcgcatgaccaacttcgacaagaacctcccgaacgagaaggtgctcccgaagcactcactcctctacgagtacttcaccgtgtacaacgagctgaccaaggtgaagtacgtgaccgaggggatgaggaagccagctttccttagcggcgagcaaaagaaggccatcgtcgacctgctgttcaagaccaaccgcaaggtgaccgtgaagcagctcaaggaggactacttcaagaaaatcgagtgcttcgactccgtcgagatctccggcgtcgaggataggttcaatgcctccctcgggacctaccacgacctcctcaagattatcaaggacaaggacttcctcgacaacgaggagaacgaggacatcctcgaggacatcgtgctcaccctcaccctcttcgaggaccgcgagatgatcgaggagcgcctcaagacatacgcccacctcttcgacgacaaggtgatgaagcagctgaagcgcaggcgctataccggctggggcaggctctctaggaagctcatcaacggcatccgcgacaagcagtccggcaagacgatcctcgacttcctcaagtccgacggcttcgccaaccgcaacttcatgcagctcatccacgacgactccctcaccttcaaggaggacatccaaaaggcccaggtgtccggccaaggcgattccctccatgaacatatcgccaatctcgccggctccccggctatcaagaagggcattctccagaccgtgaaggtggtggacgagctggtgaaggtgatgggcaggcacaagccagagaacatcgtgatcgagatggcccgcgagaaccagaccacacagaagggccaaaagaactcccgcgagcgcatgaagaggatcgaggagggcattaaggagctgggctcccagatcctcaaggagcacccagtcgagaacacccagctccagaacgagaagctctacctctactacctccagaacggccgcgacatgtacgtggaccaagagctggacatcaaccgcctctccgactacgacgtggacGCtattgtgccgcagtccttcctgaaggacgactccatcgacaacaaggtgctcacccgctccgacaagaacaggggcaagtccgataacgtgccgtccgaagaggtcgtcaagaagatgaagaactactggcgccagctcctcaacgccaagctcatcacccagaggaagttcgacaacctcaccaaggccgagagaggcggcctttccgagcttgataaggccggcttcatcaagcgccagctcgtcgagacacgccagatcacaaagcacgtggcccagatcctcgactcccgcatgaacaccaagtacgacgagaacgacaagctcatccgcgaggtgaaggtcatcaccctcaagtccaagctcgtgtccgacttccgcaaggacttccagttctacaaggtgcgcgagatcaacaactaccaccacgcccacgacgcctacctcaatgccgtggtgggcacagccctcatcaagaagtacccaaagctcgagtccgagttcgtgtacggcgactacaaggtgtacgacgtgcgcaagatgatcgccaagtccgagcaagagatcggcaaggcgaccgccaagtacttcttctactccaacatcatgaatttcttcaagaccgagatcacgctcgccaacggcgagattaggaagaggccgctcatcgagacaaacggcgagacaggcgagatcgtgtgggacaagggcagggatttcgccacagtgcgcaaggtgctctccatgccgcaagtgaacatcgtgaagaagaccgaggttcagaccggcggcttctccaaggagtccatcctcccaaagcgcaactccgacaagctgatcgcccgcaagaaggactgggacccgaagaagtatggcggcttcgattctccgaccgtggcctactctgtgctcgtggttgccaaggtcgagaagggcaagagcaagaagctcaagtccgtcaaggagctgctgggcatcacgatcatggagcgcagcagcttcgagaagaacccaatcgacttcctcgaggccaagggctacaaggaggtgaagaaggacctcatcatcaagctcccgaagtacagcctcttcgagcttgagaacggccgcaagagaatgctcgcctctgctggcgagcttcagaagggcaacgagcttgctctcccgtccaagtacgtgaacttcctctacctcgcctcccactacgagaagctcaagggctccccagaggacaacgagcaaaagcagctgttcgtcgagcagcacaagcactacctcgacgagatcatcgagcagatctccgagttctccaagcgcgtgatcctcgccgatgccaacctcgataaggtgctcagcgcctacaacaagcaccgcgataagccaattcgcgagcaggccgagaacatcatccacctcttcaccctcaccaacctcggcgctccagccgccttcaagtacttcgacaccaccatcgaccgcaagcgctacacctctaccaaggaggttctcgacgccaccctcatccaccagtctatcacaggcctctacgagacacgcatcgacctctcacaactcggcggcgatAGTGGTTCTGAAACTCCTGGAACATCAGAATCGGCGACGCCGGAGAGCGCTACCGTCGTGTCCGGGCAGAAACAAGATAGACAAGGCGGCGAGAGGAGGCGTAGCCAGCTGGACCGCGACCAGTGCGCCTACTGCAAGGAGAAGGGCCACTGGGCAAAAGATTGTCCAAAGAAGCCCCGCGGGCTAAGAGGACCTCGGCCGCAGACCTCCCTCCTTTCAGGCGGCTCCAGCGGCGGCTCTAAGCGGACCGCCGACGGATCAGAGTTCGAGAGCCCGAAGAAGAAGAGGAAGGTGTCCGGCGGCTCATCTGGCGGCTCCACACTCAATATCGAGGACGAGTACAGGCTGCATGAGACATCCAAGGAGCCTGACGTCTCCCTCGGCAGCACATGGCTCTCAGATTTCCCACAGGCCTGGGCCGAGACAGGCGGCATGGGCCTCGCCGTCCGCCAGGCGCCGCTCATCATTCCACTGAAGGCGACCTCCACACCGGTGAGCATCAAGCAGTACCCAATGTCTCAGGAGGCAAGGCTGGGCATCAAGCCACACATTCAGAGGCTCCTGGACCAGGGCATTCTGGTGCCTTGCCAGAGCCCGTGGAACACCCCTCTCCTGCCGGTGAAGAAGCCTGGCACAAATGACTACCGCCCGGTCCAGGATCTCAGGGAGGTGAACAAGCGCGTCGAGGATATCCATCCGAacGTCCCGAACCCATACAATCTCCTGTCAGGCCTCCCGCCATCTCACCAGTGGTACACCGTGCTCGACCTGAAGGATGCGTTCTTCTGCCTCAGGCTGCATCCAACAAGCCAGCCTCTCTTCGCCTTCGAGTGGCGCGATCCGGAGATGGGCATTTCAGGCCAGCTCACCTGGACACGGCTGCCACAGGGCTTCAAGAACTCTCCTACCCTCTTCtgTGAGGCGCTCCATCGGGACCTGGCCGATTTCAGGATCCAGCACCCAGACCTCATTCTCCTCCAGTATtatGACGATCTCCTGCTCGCCGCGACATCCGAGCTGGATTGCCAGCAGGGAACCCGCGCGCTGCTCCAGACACTGGGAAATCTGGGATACAGGGCATCAGCGAAGAAGGCACAGATCTGCCAGAAGCAGGTCAAGTACCTCGGCTACCTGCTCAAGGAGGGACAGAGGTGGCTGACAGAGGCAAGGAAGGAGACAGTGATGGGCCAGCCTACCCCGAAGACACCACGGCAGCTCAGGGAGTTCCTGGGCAAGGCGGGCTTCTGCCGCCTCTTCATCCCAGGATTCGCGGAGATGGCGGCGCCACTCTACCCTCTGACCAAGCCTGGCACACTGTTCAACTGGGGACCAGACCAGCAGAAGGCGTACCAGGAGATTAAGCAGGCCCTGCTCACAGCACCTGCCCTCGGCCTGCCGGACCTCACAAAGCCATTCGAGCTGTTCGTGGATGAGAAGCAGGGCTACGCGAAGGGAGTCCTGACACAGAAGCTGGGACCATGGAGGCGCCCAGTGGCCTACCTCTCCAAGAAGCTGGACCCAGTGGCTGCCGGCTGGCCTCCGTGCCTGAGGATGGTGGCGGCCATTGCCGTCCTCACCAAGGATGCCGGCAAGCTGACAATGGGCCAGCCTCTCGTCATTCTGGCGCCGCATGCGGTGGAGGCGCTCGTCAAGCAGCCACCTGATAGGTGGCTGTCCAACGCGCGCATGACCCACTACCAGGCCCTGCTCCTGGACACAGATAGGGTGCAGTTCGGCCCAGTGGTCGCCCTCAATCCTGCCACACTGCTGCCACTCCCTGAGGAGGGCCTCCAGCATAACTGCCTCGATATTCTGGCGGAGGCCCATGGAACCCGCCCTGACCTCACAGATCAGCCGCTGAGCCCGAAGAAGAAGAGGAAAGTGGGATCAGGACCAGCCGCCAAGAGGGTGAAGCTCGATTAG

Blue: SpCas9 nickase;

Red: NC peptide;

Green: M-MLV RT variant;

Red: NLS.

32
